# Sex differences in the effects of individual anxiety state on regional responses to negative emotional scenes

**DOI:** 10.21203/rs.3.rs-3701951/v1

**Published:** 2023-12-19

**Authors:** Shefali Chaudhary, Hak Kei Wong, Yu Chen, Sheng Zhang, Chiang-Shan R. Li

**Affiliations:** Yale School of Medicine: Yale University School of Medicine; University College London; Yale School of Medicine: Yale University School of Medicine; Yale School of Medicine: Yale University School of Medicine; Yale University School of Medicine

**Keywords:** sex difference, negative emotion, anxiety, fMRI, Hariri

## Abstract

**Background:**

Men and women are known to show differences in the incidence and clinical manifestations of mood and anxiety disorders. Many imaging studies have investigated the neural correlates of sex differences in emotion processing. However, it remains unclear how anxiety might impact emotion processing differently in men and women.

**Method:**

We recruited 119 healthy adults and assessed their levels of anxiety using State-Trait Anxiety Inventory (STAI) State score. With functional magnetic resonance imaging (fMRI), we examined regional responses to negative vs. neutral (Neg-Neu) picture matching in the Hariri task. Behavioral data were analyzed using regression and repeated-measures analysis of covariance with age as a covariate, and fMRI data were analyzed using a full-factorial model with sex as a factor and age as a covariate.

**Results:**

Men and women did not differ in STAI score, or accuracy rate or reaction time (RT) (Neg-Neu). However, STAI scores correlated positively with RT (Neg-Neu) in women but not in men. Additionally, in women, STAI score correlated positively with lingual gyrus (LG) and negatively with medial prefrontal cortex (mPFC) and superior frontal gyrus (SFG) activity during Neg vs. Neu trials. The parameter estimates (β’s) of mPFC also correlated with RT (Neg-Neu) in women but not in men. Generalized psychophysiological interaction (gPPI) analysis in women revealed mPFC connectivity with the right inferior frontal gyrus, right SFG, and left parahippocampal gyrus during Neg vs. Neu trials in positive correlation with both STAI score and RT (Neg-Neu). In a mediation analysis, mPFC gPPI but not mPFC activity fully mediated the association between STAI scores and RT (Neg-Neu).

**Conclusion:**

With anxiety affecting the behavioral and neural responses to negative emotions in women but not in men and considering the known roles of the mPFC in emotion regulation, we discussed heightened sensitivity and regulatory demands during negative emotion processing as neurobehavioral markers of anxiety in women.

## Plain Language Summary

Men and women often experience and express their emotional problems in different ways. In this study, we investigated how anxiety affects negative emotion processing in men and women. By understanding these differences, we hope to elucidate how men and women differ in the perception and processing of negative emotions in association with individual differences in anxiety. To this end, we recruited 60 men and 59 women from the community. We evaluated participants’ anxiety state using a validated instrument and their brain responses to negative emotional and neutral pictures in picture matching task using functional brain imaging. The results showed that individual levels of anxiety were positively correlated with the speed of matching negative vs. neutral pictures, suggesting interference of negative emotions with cognitive motor processing, in women, but not in men. Thus, women with more severe anxiety may be more sensitive to distraction by negative emotional stimuli. In brain imaging data, the activities of the medial prefrontal cortex, a region that supports emotion regulation, during negative vs. neutral emotion processing were negatively correlated with anxiety in women, and this effect was not seen in men. Further, the medial prefrontal cortex showed connectivities with other brain regions and these functional connectivities mediated the effects of anxiety on matching speed in women. These findings suggest that heightened sensitivity to negative emotions in anxious women are possibly due to emotion dysregulation within the medial prefrontal cortex. These findings may help us better understand why women are more vulnerable to emotional problems and develop more personalized treatments for anxiety and mood disorders.

## 1. Introduction

### 1.1 Sex differences in anxiety and mood disorders and the neurobiology of stress response

The US National Institute of Mental Health reports a 60% higher lifetime prevalence of anxiety disorders in women compared to men, and highlights sex differences in the onset, severity, clinical course, and treatment response in anxiety disorders [1–3]. Women seem to experience more severe and longer-lasting symptoms of anxiety than men [4, 5]. In a sample of over 20,000 adults, the lifetime and 12-months male to female prevalence ratios of anxiety disorder were 1:1.7 and 1:1.8, respectively, with women having higher rates of lifetime diagnosis of most anxiety disorders [6]. Further, women with a lifetime diagnosis of an anxiety disorder were more likely than men to be also diagnosed with another anxiety disorder and major depressive disorder [6] .

Both preclinical and human studies have examined the neurobiological mechanisms underlying sex differences in anxiety-like behavior. For instance, activation of the endocannabinoid 2-arachidonoyl glycerol, a key regulator of neurotransmitter release, via the cannabinoid receptor (CB1) resulted in more frequent freezing behavior in male rats, but less freezing and more frequent darting (active avoidance) in female rats [7]. In female rats and humans, fluctuations in estradiol levels can impact limbic circuit activity and fear extinction [8, 9]. Individuals with mood disorders often exhibit hypersecretion of corticotropin releasing factor (CRF), which stimulates noradrenaline release from the locus coeruleus (LC), leading to higher levels of alertness and anxiety symptoms [7]. Importantly, animal studies showed that LC neurons are more sensitive to CRF in females than in males [10, 11]. Following exposure to social stress, a single dose of intranasal oxytocin reduced distress in men but elevates distress and anger in women [12]. In animal models of social distress, blocking oxytocin receptors in the bed nucleus of the stria terminalis reduces anxiety-like behavior in female but not male mice; in contrast, oxytocin receptor blocking enhanced social-avoidance like behavior in unstressed males [13]. Together, ample evidence suggests that stress response is not only mediated through distinct neurobiological pathways but also manifested differently in behaviors between sexes.

### 1.2 Sex differences in neural processing of negative emotion

Many human imaging studies have reported differences in regional activities in viewing negative emotional vs. neutral pictures, with the amygdala, thalamus, dorsal/ventral visual cortex, parietal cortex, inferior frontal gyrus, insula, orbitofrontal and medial frontal cortices, amongst others, showing higher activity during exposure to negative emotions [14–16]. Earlier reviews and meta-analyses indicated that women generally show stronger neural responses to negative emotions, whereas men exhibit greater responses to positive emotions, in behavioral paradigms aimed to elicit emotional experiences [17, 18]. The amygdala, thalamus, caudate, putamen, superior/middle frontal gyri, and orbitofrontal gyrus showed higher responses to negative emotions in women vs. men, whereas the amygdala, inferior frontal gyrus, and fusiform gyrus showed higher responses to positive emotions in men vs. women [17, 18]. However, in a later meta-analysis, no differences between men and women was noted during negative vs neutral emotion processing [19]. David and colleagues identified no significant increase in the number of regional foci with larger sample sizes, suggesting the presence of excess “significance bias,” i.e., reporting bias, in the neuroimaging literature on sex differences [20]. Further, a recent meta-analysis did not observe significant effects of sex in meta-regression of negative vs neutral face processing [21]. Thus, we need more studies of large sample size to revisit sex differences in negative emotion processing.

Another dimension of sex differences concerns the correlates of individual variation. A few studies noted no sex differences in overall brain activity but significant differences in the neural correlates of individual variation in subjective experiences, including arousal [22], anxiety [23], and mood [24] ratings during negative emotion processing. These findings highlight a critical dimension of sex differences that have not been thoroughly explored. Further, previous imaging studies have either employed a paradigm that required no explicit behavioral response or have not examined sex differences in neural correlates of behavioral performance. This contrasts with animal studies where anxiety-like behavior can be objectively quantified, as reviewed earlier. Characterizing how negative emotions may interfere with target identification in the Hariri task (valenced/neutral picture matching task [25]), for instance, would offer a behavioral measure of individual variation in anxiety and a venue to investigate sex differences in the impact of anxiety on negative emotion processing.

### 1.3 Anxiety and negative emotion processing

Emotional states can alter how we process affective stimuli, as noted in many studies of people with mood disorders. For instance, compared to healthy controls, individuals with social anxiety disorder exhibited higher bilateral amygdala and insula activity during identification of negative vs. neutral images [26]. Another study noted greater left amygdala and inferior frontal gyrus activation in individuals with generalized anxiety disorder, as compared to healthy participants, viewing emotionally negative vs. neutral pictures [27]. A meta-analysis of individuals with social anxiety, post-traumatic stress disorder, and specific phobia showed hyperactive amygdala and insula during passive viewing or identification of negative vs. positive or neutral emotional images or vs. a resting baseline [28]. Individuals with anxiety disorders relative to neurotypical people showed higher right anterior insula activation and connectivity with frontoparietal regions during anticipatory anxiety [29]. Individuals with anxiety and mood disorders exhibited higher amygdala and visual cortical responses to passively viewing negative, emotionally arousing scenes, such as those involving violence or contamination, as compared to neutral scenes [30]. Furthermore, lower reactivity in these regions while viewing emotional as opposed to neutral scenes was correlated with higher trauma scores, suggesting blunted neural activities in response to more severe and oftentimes repeated exposure to trauma [30].

Apart from mood disorders, individual variation in anxiety can influence how emotional stimuli are processed in neurotypical populations. Individuals with higher elevation in cortisol levels (greater stress response) showed lower OFC activity during negative vs neutral emotion processing [31]. In another study, ventromedial prefrontal cortical activity during threat vs. safe condition increased with greater individual state of anxiety [32]. A few studies reported the findings in women or men alone or specifically noted sex differences in the findings of individual variation. For instance, the severity of dysphoric mood, as assessed through the Profile of Mood States and State-Trait Anxiety Inventory, was associated with heightened hypothalamic activity during the processing of negative vs. neutral images [24]. The latter study also reported elevated amygdala activity in positive correlation with dysphoric mood in women but not in men [24]. In contrast, a more recent work noted retro-splenial cortex and precuneus activity during negative emotional face vs neutral shape identification in negative correlation with NIH Toolbox anger- and fear-affect scores in men but not in women [33]. Thus, these studies indicate that anxiety’s impact on negative emotions may manifest in a sex-specific manner, emphasizing the need for further exploration in this direction.

Together, earlier studies demonstrate the impact of individual differences in mood and anxiety, whether meriting a clinical diagnosis or not, on the neural activities of negative emotion processing. Here, we aimed to study how such an impact of individual differences in mood and anxiety may vary between men and women.

### 1.4 The present study

We recruited 119 healthy adults, evaluated their anxiety state with the State-Trait Anxiety Inventory, and tested their brain responses to negative emotion in a Hariri picture matching task [25] using International Affective Picture System (IAPS, a database of pictures for studying emotion) negative and neutral pictures. A widely used paradigm to query brain activation to negative emotional stimuli, the Hariri task reliably engages corticolimbic structures [25, 34, 35].

We have two distinct aims. First, we revisited sex differences in regional brain activations during negative emotion processing. As the latest meta-analyses suggested no sex differences in the overall brain responses, we hypothesized no sex differences between men and women in their regional responses to matching of pictures of negative vs. neural emotional content. Second, we examined sex differences in the influences of individual anxiety state on both the behavioral performance and neural responses to negative emotion processing. Accurate and expedient matching in the Hariri task would require participants to divert their attention away from their natural emotional reactions and concentrate on generating a motor response. Thus, a faster reaction time (RT) would indicate better emotion regulation and less reactivity [36]. We posited that individuals with higher levels of anxiety would be more sensitive to the interference by negative emotional stimuli on cognitive motor processing and demonstrate prolonged RT and diminished activities in the emotion regulatory circuit in matching negative vs. neutral pictures. Further, this effect would be more prominent in women than in men. Finally, we performed mediation analyses to characterize the inter-relationship of individual anxiety, regional brain activities, and RT.

## 2. Methods

### 2.1 Participants and clinical assessments

One hundred and nineteen healthy adults (59 women) 19 to 85 years of age volunteered to participate in the study. Candidates were recruited from the greater New Haven, Connecticut, area. All participants were physically healthy, cognitively intact (Mini Mental State Examination Score > 27) with no major medical conditions. Those with current use of prescription medications or with a history of head injury or neurological illness were excluded. Other exclusion criteria included current or history of Axis I disorders according to the Structured Clinical Interview for DSM-IV [37]. Candidates who reported current use of illicit substances or tested positive for cocaine, methamphetamine, opioids, marijuana, barbiturates, or benzodiazepines were not invited to participate. All participants were assessed with the State-Trait Anxiety Inventory (STAI). The STAI State score ranged from 20 to 63 with a mean ± SD of 32.24 ± 10.41 in the current sample. The Human Investigation Committee at Yale School of Medicine approved the study procedures. All participants signed an informed consent prior to the study.

### 2.2 MRI protocol and behavioral task

Brain images were collected using multiband imaging with a 3-Tesla MR scanner (Siemens Trio, Erlangen, Germany). Conventional T1-weighted spin echo sagittal anatomical images were acquired for slice localization. Anatomical 3D MPRAGE image were next obtained with spin echo imaging in the axial plane parallel to the AC–PC line with TR = 1900 ms, TE = 2.52 ms, bandwidth = 170 Hz/pixel, field of view = 250 × 250 mm, matrix = 256 × 256, 176 slices with slice thickness = 1 mm and no gap. Functional, blood oxygen level-dependent (BOLD) signals were acquired with a single-shot gradient echoplanar imaging sequence. Fifty-one axial slices parallel to the AC–PC line covering the whole brain were acquired with TR = 1000 ms, TE = 30 ms, bandwidth = 2290 Hz/pixel, flip angle = 62°, field of view = 210 × 210 mm, matrix = 84 × 84, 51 slices with slice thickness = 2.5 mm and no gap, 392 volumes, and multiband acceleration factor = 3. Images from the first ten TRs at the beginning of each scan were discarded to ensure that only BOLD signals in steady-state equilibrium between RF pulsing and relaxation were included in data analyses.

**Figure 1:**
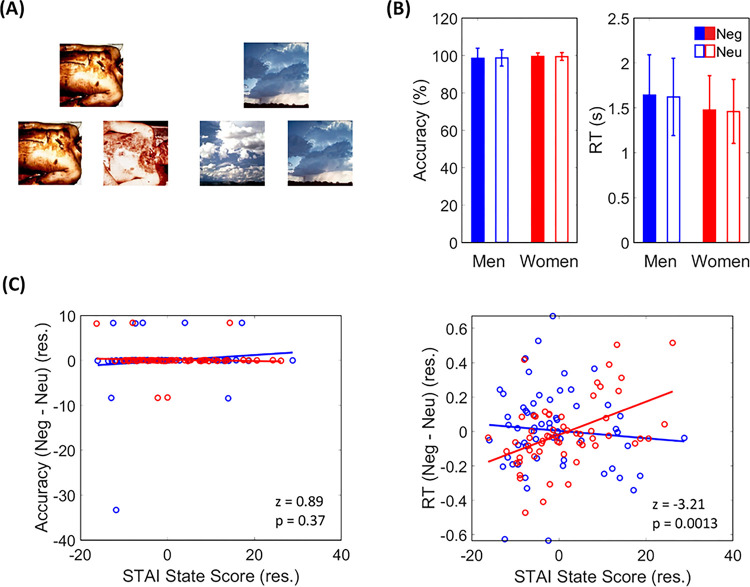
Behavioral task and performance. (A) Example images used in the matching task. (B) Accuracy rate and reaction time (RT) plotted separately for men and women. (C) Correlation of difference in accuracy rate and of RT between negative and neutral blocks with anxiety scores. Note: Data points representing men and women are shown in blue and red, respectively.

In the Hariri picture matching task, 24 different images were used, with 12 each of negative and neutral emotional IAPS pictures, in a block design. The target picture was shown on the top and two pictures either matching or not matching the target were shown at the bottom. Participants were asked to match one of two simultaneously presented pictures with the target picture by pressing a left or right buttons on their right or dominant hand (Fig. 1A). A session comprised 10s of dummy scans, followed by the task instruction to “choose one to match the picture at the top” for 2s and 4 picture blocks in the sequence: one neutral block ◊ two negative blocks ◊ one neutral block. Each block started with a fixation period of 2s, followed by 6 stimuli each lasting 6s. The 6 stimuli were presented consecutively without inter-stimuli gap. The blocks last approximately 152s (2.5 minutes). During imaging, subjects responded by pressing one of two buttons, allowing for the determination of accuracy and reaction time (RT). Subjects were told that the stimuli would be presented long enough for them to make an accurate match but were not explicitly instructed to respond as fast as possible. This allowed us to assess the natural preferences in emotion processing across subjects [38]. Please note that this task is a component of a larger task, and we focused on the picture matching blocks in the current manuscript.

### 2.3 Imaging data processing and modeling

Data were analyzed with Statistical Parametric Mapping (SPM12, Welcome Department of Imaging Neuroscience, University College London, U.K.), following our published routines [36]. Images of each individual subject were first realigned (motion corrected) and corrected for slice timing. A mean functional image volume was constructed for each subject per run from the realigned image volumes. These mean images were co-registered with the high-resolution structural image and segmented for normalization with affine registration followed by nonlinear transformation. The normalization parameters determined for the structure volume were then applied to the corresponding functional image volumes for each subject. The resampled voxel size is 2.5 × 2.5 × 2.5 mm^3^. Finally, the images were smoothed with a Gaussian kernel of 8 mm at Full Width at Half Maximum.

A statistical analytical block design was constructed for each individual subject using a general linear model (GLM) by convolving the canonical hemodynamic response function (HRF) with the boxcar function in SPM, separately for negative and neutral images. Realignment parameters in all six dimensions were also entered in the model. The GLM estimated the component of variance that could be explained by each of the regressors.

### 2.4 Statistical analyses of imaging data

In the first-level analysis, we constructed for each individual subject a contrast of negative vs. neutral picture blocks (Neg-Neu) to evaluate differences in regional responses to matching these images. The contrast images (.*con*) of the first-level analysis were used for group statistics. In random effects analyses, we conducted a full-factorial analysis on all subjects’ .*con*, with sex as a two-level factor, STAI score as a covariate with interaction effects involving sex, and age as a covariate of no interest (SPM design matrix shown in **Supplementary Figure S1**). The model factored the STAI score based on sex and enabled us to evaluate differences in the regression slope of (Neg-Neu) activity against STAI score between men and women, controlling for the overall effect of age [39]. We assessed the model for: (1) BOLD activity during (Neg-Neg) in men, women and all participants and differences in BOLD activity between men and women (men > women, women > men) (2) regression slope differences in BOLD activity during (Neg-Neu) against STAI score between men and women, as well as regression separately in men and women, using T-contrasts. Following current reporting standards [36], all results were evaluated with voxel p < 0.001, uncorrected, in combination with cluster p < 0.05, FWE corrected, on the basis of Gaussian random field theory as implemented in SPM.

We used MarsBaR (http://marsbar.sourceforge.net/) to derive for each individual subject the parameter estimates (β’s) of the functional ROIs identified from full factorial analysis and assessed the correlation between β’s and behavioral data. In addition to whole-brain analyses of a directional contrast of men and women in STAI score regression, we performed slopes tests to examine sex differences in the regression of β’s identified of men or women alone vs. STAI score. As a threshold was imposed in whole-brain regressions and those findings identified in, say, women, might have just missed the threshold in men, and vice versa. Thus, a slope test was needed to confirm sex differences, an analysis that should not be considered as “double-dipping.”

### 2.5. Connectivity analysis: Psychophysiological interaction (PPI)

We conducted a generalized gPPI analysis with significant clusters identified from whole-brain correlates of STAI score (See Results) to explore anxiety-related changes in functional connectivity during emotion processing. Following published methods [36], we created a PPI model for each subject with three regressors: the physiological variable that represents temporally filtered, mean-corrected and deconvolved time series of the seed region, the psychological variable that represents the task contrast (negative vs. neutral), and a PPI variable that was computed as element-by-element product of deconvolved time series of the seed and contrast, followed by re-convolution with the HRF. The PPI images of each subject were used in random effect analyses – including whole-brain regression against STAI score and RT (Neg-Neu).

With MarsBaR, we extracted the average functional connectivity (FC β) between the seed and clusters (if any) identified from regression analysis and assessed the correlations between the FC β’s and behavioral data.

### 2.6 Mediation analyses

For the clusters with activity and/or connectivity (FC) β’s correlated both with STAI score and RT, we performed mediation analyses, with ‘age’ as covariate to characterize the inter-relationships of these clinical, behavioral, and neural metrics (see Results), following our previous study [40] and as described in the Supplement. We specifically focused on the model: [anxiety → β/FC β → RT] to test the hypotheses that the neural correlates mediated the effects of anxiety on behavioral performance.

## 3. Results

### 3.1 Behavioral results:

Across negative and neutral trials, the mean RTs ranged from 0.82 to 3.16 s and the mean accuracy rates ranged from 71 to 100% across subjects (Fig. 1B). A 2 (stimulus: negative vs. neutral) × 2 (sex: men vs. women) ANOVA with age as a covariate did not show any significant main or interaction effects for accuracy rate: main stimulus effect (F_1,117_ = 0.00, p = 0.997), main sex effect (F_1,117_ = 2.45, p = 0.120), stimulus × sex (F_1,117_ = 0.14, p = 0.708); or for RT: main stimulus effect (F_1,117_ = 0.68, p = 0.411), main sex effect (F_1,117_ = 0.01, p = 0.910), stimulus × sex (F_1,117_ = 3.65, p = 0.058).

Controlling for age, men and women did not differ in the STAI score (men: 30.37 ± 10.22, women: 34.15 ± 10.34, p = 0.137). Neither accuracy rate (Neg – Neu) or RT (Neg – Neu) showed a significant correlation with the STAI score in Pearson regression with age as a covariate: accuracy rate (r = 0.06, p = 0.506) and RT (r = 0.15, p = 0.095) for all subjects; accuracy rate (r = 0.11, p = 0.385) and RT (r = −0.08, p = 0.517)) for men. In women, RT (Neg-Neu) but not the accuracy rate (Neg – Neu) showed a significant correlation with STAI score (r = 0.48, p < 0.001 and r = −0.05, p = 0.699, respectively). Slope test revealed significant differences in regression slope of RT vs. STAI score (t = 3.20, p = 0.002) but not of accuracy rate vs. STAI score (t = −0.66, p = 0.509). These findings are shown in Fig. 1C and 1D. Thus, although the behavioral performance in matching negative vs. neutral pictures did not vary between men and women, anxiety significantly affected performance in women but not in men.

### 3.2 Imaging results:

#### 3.2.1 Neural responses to matching of negative vs. neutral pictures

Across all subjects, bilateral inferior occipital gyrus, superior frontal gyrus, middle/inferior frontal gyrus, left amygdala, and left thalamus/caudate showed higher activation during matching of negative vs. neutral pictures (**Supplementary Fig. 2A**). This pattern of activation was consistent in men (**Supplementary Fig. 2B**) and women (**Supplementary Fig. 2C**). Although women appeared to show greater regional activations than men, the differences were not significant in a direct contrast.

#### 3.2.2 Neural correlates of anxiety

**Figure 2:**
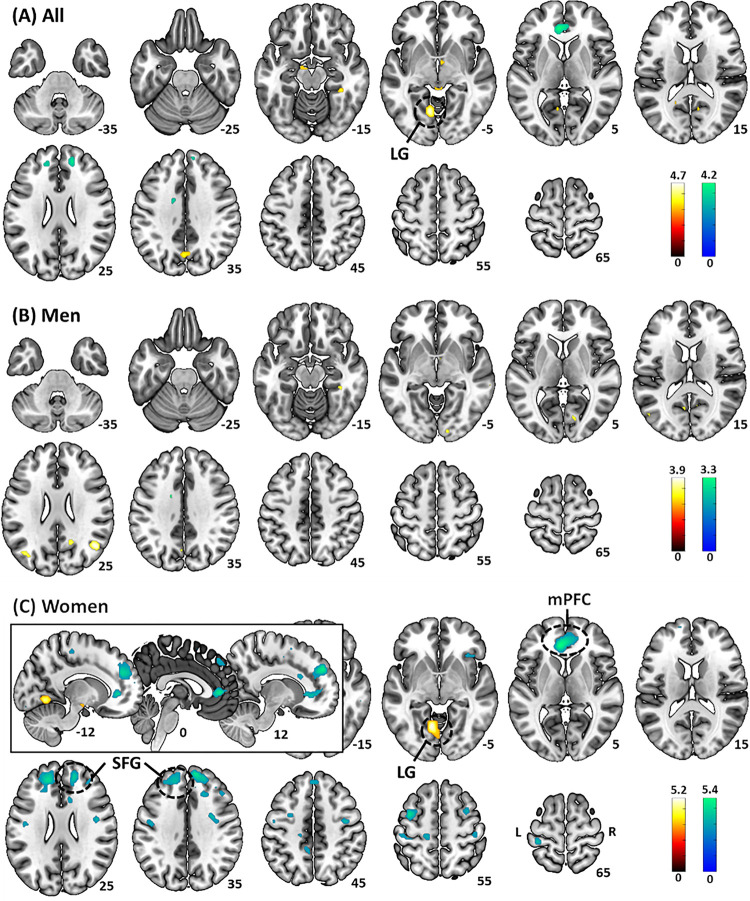
Whole-brain regression of the contrast (Neg – Neu) against STAI score with age as a covariate in (A) all subjects, (B) men, and (C) women, evaluated at p<0.001, uncorrected. Color bars show voxel T values, with warm and cool color each for positive and negative correlation. LG: lingual gyrus; mPFC: medial prefrontal cortex; SFG: superior frontal gyrus. The inset in (C) showed the mPFC cluster in sagittal sections.

In whole-brain regression of (Neg-Neu) activity against STAI score with age as a covariate, a single cluster in the lingual gyrus (LG, x= −10, y= −64, z= −7, voxel Z = 4.50, 139 voxels) showed activity in positive correlation with STAI score across all subjects (Fig. 2A). The analyses in men alone did not reveal any significant clusters (Fig. 2B). In women alone, a cluster in the LG (x= −10, y= −61, z= −7, voxel Z = 4.88, 150 voxels) showed activity in positive correlation with STAI score, and three clusters each in the medial prefrontal cortex (mPFC, in pregenual and subgenual anterior cingulate gyrus; x = −8, y = 36, z = 3, voxel Z= −5.11, 295 voxels), right superior frontal gyrus (SFG, x = 15, y = 46, z = 28, voxel Z=−4.77, 262 voxels), and left SFG (x= −15, y = 42, z = 28, voxel Z= −4.61, 354 voxels) showed activity in negative correlation with STAI score (Fig. 2C). We did not observe any clusters showing significant sex differences in the regression of (Neg-Neu) activity against STAI score in whole-brain analysis.

We extracted the β estimates of (Neg-Neu) of the LG cluster identified from the regression across all subjects. The β’s were correlated significantly with the STAI score (r = 0.37, p < 0.001), as expected, and also significantly with the RT (Neg-Neu) but not accuracy rate (Neg-Neu), with age as covariate (r = 0.32, p < 0.001 and r= −0.11, p = 0.247, respectively). In a slope test, men and women did not differ significantly in regression slope of LG vs. STAI score (t= −1.47, p = 0.144) or vs. RT (Neg-Neu), with age as covariate (t = 1.73, p = 0.086).

We also extracted the β’s of “Neg-Neu” of the LG, mPFC, and SFG clusters identified in women. With age as a covariate, the clusters showed β’s in significant correlation with the STAI score in women, as expected: LG (r = 0.45, p < 0.001), mPFC (r = −0.45, p < 0.001), right SFG (r = −0.51, p < 0.001), and left SFG (r = −0.49, p < 0.001). In slope tests with age as a covariate, men and women showed significant differences in regression slope of the β’s vs. STAI score for the mPFC (t = −3.17, p = 0.002), right SFG (t = −2.76, p = 0.007), left SFG (t = −3.11, p = 0.002), and marginally for the LG (t = 2.13, p = 0.035).

We evaluated the relationship of these β’s and RT (Neg-Neu) and accuracy rate (Neg-Neu) in women. The β’s of the LG (r = 0.43, p < 0.008) and mPFC (r = −0.29, p = 0.026), but not the right SFG (r = −0.23, p = 0.083) or left SFG (r = −0.18, p = 0.172) were significantly correlated with RT (Neg-Neu), with age as covariate. In slope tests of β’s vs. RT (Neg-Neu), the mPFC (t = −2.50, p = 0.014) but not the LG β (t = 1.95, p = 0.054) showed significant sex differences in the regression slope. None of the β’s was significantly correlated with accuracy rate (Neg-Neu) (−0.06 < r’s < 0.04, 0.676 < p’s < 0.991).

To summarize, for all of the clusters identified from whole-brain regression against STAI score across all subjects or in women alone, only the mPFC cluster identified from women showed a significant correlation of the β’s with RT (Neg-Neu) as well as a significant sex difference in slope in the regression of the β’s vs. STAI score and of the β’s vs. RT (Neg-Neu).

#### 3.2.3 Functional connectivity

**Figure 3:**
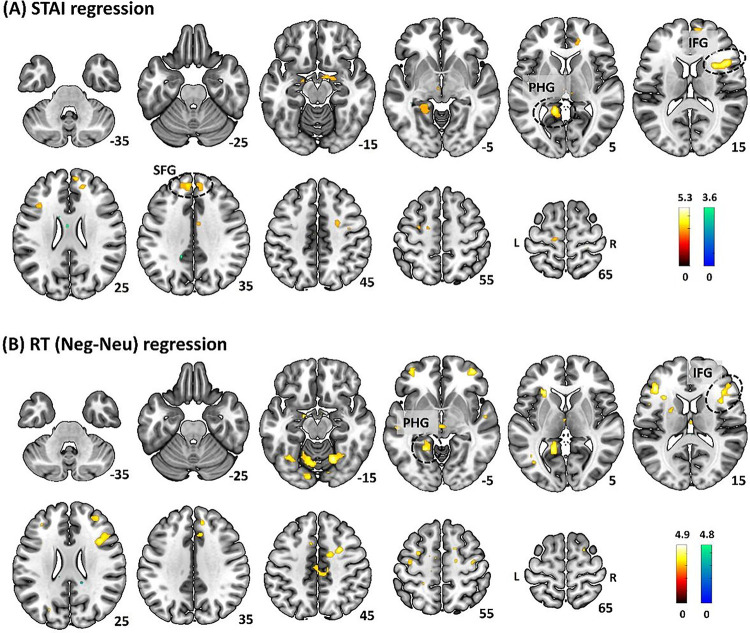
Whole-brain mPFC gPPI regression on (A) STAI score and (B) “neg-neu” RT in women. The gPPI seeds are shown in ‘Red’; IFG: inferior frontal gyrus, SFG: superior frontal gyrus, PHG: parahippocampal gyrus. Color bars show voxel T values, with warm and cool color each for positive and negative correlations.

The mPFC cluster identified from women showed a significant correlation of the β’s with RT (Neg-Neu) as well as a significant sex difference in slope in the regression of the β’s vs. STAI score and of the β’s vs. RT (Neg-Neu). Thus, we focused on the mPFC cluster as a seed region and conducted a gPPI analysis. The results showed (Neg-Neu) gPPI correlates of STAI score in the right superior frontal gyrus (SFG) and inferior frontal gyrus (IFG) and left parahippocampal gyrus (PHG). The extracted gPPI β’s of these clusters (Table 1, Fig. 3A) as well as the average gPPI β (r = 0.49, p < 0.001) correlated significantly with RT (Neg - Neu). In a separate regression, we identified gPPI correlates of RT (Neg - Neu) in the PHG, and IFG. The extracted gPPI β’s of these clusters (Table 1, Fig. 3B) and the average β (r = 0.47, p < 0.001) correlated with STAI score.

### 3.3 Mediation analyses

We performed mediation analysis to assess the mediating effects of mPFC β and mPFC FC β (average of all clusters identified in gPPI regression) on the association between anxiety and RT. Thus, we tested the model with anxiety and RT each as the independent and outcome variable and β as the mediating variable, with ‘age’ as covariate. We tested the model separately for men and women.

**Figure 4:**
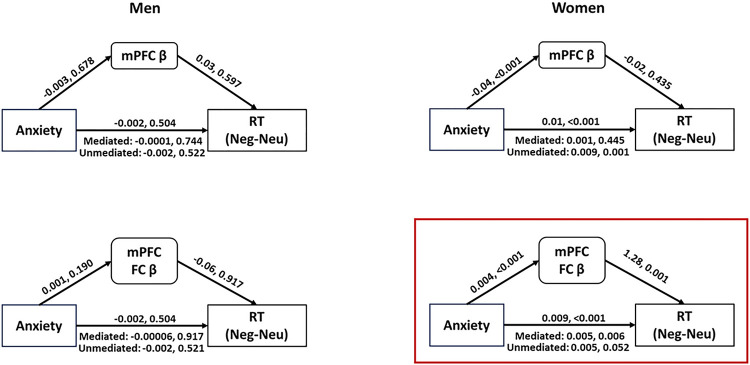
Mediation models of mPFC β/mPFC FC β, anxiety, RT (Neg-Neu), with age as covariate. Note: the path statistics represent the coefficient and p value; mPFC: middle prefrontal cortex, FC: functional connectivity, β: parameter estimate, RT: reaction time.

The model with mPFC β was not significant either in men or in women; however, the model with mPFC FC β was significant in women but not in men (Fig. 4, **Supplementary Table S1**). Thus, mPFC connectivity, but not the mPFC activity mediated the association between anxiety and RT (Neg-Neu) in women. In men, neither mPFC activity nor connectivity mediated the association between anxiety and RT (Neg-Neu).

## 4. discussion

Men and women did not demonstrate significant differences in behavioral performance in the Hariri task. However, women but not men showed a significant correlation between STAI score and RT (Neg – Neu), and the sex difference was confirmed by a slope test. Men and women also did not demonstrate significant differences in regional activities during matching negative vs. neutral images, consistent with the findings of the latest meta-analysis [19]. However, women but not men showed a significant correlation between mPFC activity and STAI score, with the sex difference confirmed by slope test. Functional connectivity revealed by gPPI analysis with the mPFC cluster as seed identified the right inferior frontal gyrus, right superior frontal gyrus and left parahippocampal gyrus with gPPI in positive correlation both with STAI score and RT (Neg – Neu). Mediation analysis described a significant model whereby STAI score influenced mPFC connectivities and in turn the RT. Together, the findings suggest sex differences in the neural and behavioral processes underlying individual differences in anxiety. Studies with other task paradigms are needed to investigate how the behavioral and neural processes of anxiety may manifest in men.

### 4.1 Behavioral correlates of anxiety

We did not observe significant differences in RT or accuracy rate (Neg-Neu) between men and women, consistent with earlier findings of no sex differences in an emotional Stroop task [41]. Similarly, a review article highlighted the lack of a clear pattern of sex differences in RT across different emotion processing tasks [42]. Note that the current findings should be considered specific to non-clinical samples, where the interference caused by emotional content may not significantly impact performance. Furthermore, although anxiety scores and RT (Neg-Neu) were both comparable between men and women, anxiety showed a positive correlation with RT (Neg-Neu) in women but not in men. This suggests that women’s response to negative emotion is more sensitive to their state of anxiety, such that higher anxiety slows the motor response, possibly due to greater attention to negative emotional content hindering task performance [36]. These findings not only characterize a behavioral correlate of anxiety in women but also suggest the importance of examining the data of men and women separately in investigating individual differences in emotion processing.

### 4.2 Neural Correlates: mPFC activity

Negative vs. neutral emotional picture processing reliably activated cotico-limbic regions in all, men, and women, with men and women showing statistically indistinguishable patterns of activations, consistent with a previous meta-analysis [19]. In women and in all subjects, we observed a positive association between anxiety and LG activity, and in women, a negative association between anxiety and mPFC and SFG activity, during negative vs. neutral processing.

A higher-order visual area, the LG is involved in processing emotional stimuli and experience [43–45]. In the present study, LG showed a trend-level decrease in activity during matching of negative vs neutral pictures (**Supplementary Figure S3**), consistent with earlier reports of reduced LG activity during negative vs neutral face/picture processing [44, 46, 47] and greater activity during happy vs neutral face processing [48]. Across all subjects and in women, LG activity correlated positively with anxiety, suggesting that LG activity elevates in participants who focus more on the negative emotional content of the pictures. Hence, we also noted longer RT with higher LG activity during matching of negative vs. neutral images, an effect that did not appear to be sex different. These findings also suggest that visual processing can be significantly affected by anxiety.

In women, we observed a negative correlation between anxiety and frontal cortical (mPFC and SFG) activation during negative vs neutral picture processing. Frontal cortical activation is noted widely across studies of emotion picture/scene processing [14–16]. Whereas the broad mPFC responds to reward and self-referential evaluation [49] as well as appraisal, regulation, and expression of emotion [50], the pregenual and subgenual anterior cingulate cortex (pgACC and sgACC) appears most critical in emotion regulation [50]. However, studies of people with anxiety disorders have reported mixed findings, with hyperactivity [51, 52], hypoactivity [53, 54] or no differences in activity [55–57] of the mPFC all been reported in individuals with general anxiety disorder (GAD) vs. controls during exposure to negative emotions. In a meta-analysis of regional responses to negative emotions, hypoactive dorsal/rostral ACC and ventromedial PFC were observed in individuals with posttraumatic stress disorder (PTSD) but not those with social anxiety disorder or specific phobia, or in healthy participants during fear conditioning [28]. Further, in an emotional Stroop task, Etkin and colleagues noted higher pgACC activity during incongruent vs. congruent trials in healthy participants but a trend of reduced activity in people with GAD [58]. Thus, literature suggests a complex pattern of anxiety-related mPFC activities during negative emotion processing that may vary with behavioral tasks and the content of anxiety. Activities of the SFG appeared to vary across behavioral tasks of emotion processing, with emotion regulation but not passive exposure eliciting higher SFG response [59–61].

A neurocognitive model posits a key role of selective attention to threat and regulation by the PFC in manifesting the effects of anxiety [62]. Here, although we did not observe significant differences in mPFC or SFG activity during negative vs. neutral picture matching (**Supplementary Figure S3**), the activity correlated negatively with state of anxiety, suggesting less emotion regulation in women with higher levels of anxiety.

### 4.3 Neural Correlates: mPFC connectivity

In women, the FC of mPFC, a component of the default mode network (DMN), showed enhanced connectivity with the SFG, IFG, and parahippocampal gyrus (PHG) in link with higher individual anxiety. The DMN comprises a set of interconnected brain regions where activities tend to increase in synchrony during unfocused or internally-directed mental states, when people are at rest, recollecting the past, or contemplating the future, but decrease during goal-directed tasks [63]. Dispositional self-focus may be more significantly elevated during negative emotional scene exposure along with higher frontal cortical interconnectivity in individuals with higher levels of anxiety [64]. Mostly noted for autobiographical memory retrieval or self-directed thought during emotion processing [65], the PHG is part of a broadly defined DMN, connecting the DMN with the memory system of the medial temporal cortex [66]. A previous study reported reduced frontal-PHG connectivity during negative emotion processing in patients with major depressive disorder and discussed the finding as a marker of impaired emotion regulation [67]. Dynamic resting connectivity between the frontal cortex and PHG was also reduced in individuals with PTSD [68]. Thus, here, enhanced mPFC-PHG connectivity in individuals with higher levels of anxiety may indicate greater emotion regulation demands in neurotypical populations, although this regulatory mechanism may come apart in people with anxiety disorders.

It’s worth noting that these FCs also exhibited significant correlations with prolonged RT (Neg-Neu), indicating their behavioral relevance. Interestingly, mPFC functional connectivity, rather than activity, completely mediated the relationship between anxiety and RT (Neg-Neu). This suggests mPFC’s role in emotion regulation but only an indirect role in manifesting the behavioral outcome of anxiety. Indeed, the SFG/IFG has been implicated in both emotion [69] and cognitive motor [70, 71] processing. For instance, in an emotional Stroop task, negative vs. neutral RT correlated with activity within a cluster that included the medial and superior frontal gyri during negative vs. neutral trials [72]. Exposure to sad versus neutral stimuli was linked to delayed stop signal reaction time, suggesting interference with motor inhibition, accompanied by heightened activation of the SFG in an emotional stop signal task [73]. In another study, greater IFG activation along with prolonged RT was noted for negative vs neutral distractors in affective Stroop task [74]. Other studies noted higher PHG activity when individuals were presented with previously encountered negatively arousing vs. neutral events during a mental navigation task, possibly as an adaptive mechanism of avoidance as shown by a faster RT [75]. In another study, imitation of emotional vs non-emotional facial expression activated the PHG as well as motor cortex, amygdala, and insula [76]. Thus, broadly consistent with these previous studies, we observed the effects of anxiety on behavioral motor response through mPFC connectivites. Notably, the findings of connectivity rather activity support the mediating effects were reported in previous studies of dopamine receptor availability and working memory [77] as well as mindfulness and implicit learning [78]. Functional connectivity as revealed by gPPI may represent neural markers of individual differences that warrant more studies.

### 4.4 Limitations and conclusion

We discussed a few limitations of the study. First, we considered the effects of individual variation in natural mood rather than experimentally modulated the state of anxiety. While this approach is valuable for assessing participants’ inherent emotional tendencies, future research is required to ascertain whether these findings apply to controlled experimental conditions. Second, our participants scored from 20 to 60 out of a range of 20 to 80 in STAI score. Thus, individuals with higher STAI score may be needed to fully understand the effects of anxiety on the behavioral and neural responses to negative emotions. Third, previous studies showed that the neural correlates of negative emotion processing may depend on the stimuli, e.g., face vs. non-face, and behavioral task, e.g., whether working memory is involved [46, 79]. Therefore, the current findings should be considered as specific to matching of emotional scenes. Finally, behavioral contingencies that distinguish passive emotional exposure and active regulation of emotions within subjects are needed in future studies to better identify regulatory activities and investigate the effects of anxiety on the circuit activity.

In conclusion, women appear to be more sensitive to anxiety when processing negative information, an effect that manifests in prolonged RT in matching negative vs. neural pictures in the Hariri task. This heightened sensitivity may be mediated by dysregulated negative emotion processing in the mPFC and other brain regions connected with the mPFC. These sex-specific findings offer insights into a behavioral and neural mechanism of susceptibility of women to mood disorders.

## Figures and Tables

**Table 1:** Whole-brain mPFC gPPI regression on STAI score and RT (Neg - Neu) in women.

volume (voxels)	peak voxel (Z)	MNI coordinates (mm)			side	identified brain region	Pearson r, p-value (age as covariate)
		x	y	z			
*Regression vs. STAI score (Positive)*							*Correlation with RT (Neg-Neu)*
144	4.73	30	12	13	R	IFG	0.43, < 0.001
	4.25	45	17	16			
114	4.17	8	59	18	R	SFG	0.31, 0.017
	3.83	13	44	28			
	3.61	8	49	33			
104	4.12	−15	−46	6	L	PHG	0.47, <0.001
	3.43	−20	−39	−5			
*Regression vs. STAI score (Negative)*							
None							
*Regression vs. RT (Neg - Neu) (Positive)*							*Correlation with STAI score*
204	4.29	−18	−51	1	L	PHG	0.41, 0.001
	4.08	−8	−69	−17			
	3.69	−13	−61	−15			
169	4.12	38	17	23	R	IFG	0.42, < 0.001
	3.83	40	24	18			
	3.82	38	14	13			
*Regression vs. RT (Neg - Neu) (Negative)*							
None							

Note: IFG: inferior frontal gyrus, SFG: superior frontal gyrus, PHG: parahippocampal gyrus

## Data Availability

Data sets are available from the corresponding author upon reasonable request.

## Highlights

- Men and women did not differ in accuracy or RT during matching of negative vs. neutral images in Hariri picture matching task.
- In women, but not in men, anxiety correlated positively with negative vs. neutral RT.
- Negative vs. neutral image matching engaged corticolimbic regions comparably in men and women.
- In women but not in men, activity of the mPFC during negative vs. neutral image matching correlated negatively with anxiety and with negative vs. neutral RT.
- In women, mPFC connectivity with the frontal cortex and parahippocampus mediated the association between anxiety and negative vs. neutral RT.
- MPFC dysfunction and heightened sensitivity to negative emotions may explain higher susceptibility of women to mood and anxiety disorders.

## Perspectives and significance

Our finding suggests that state of anxiety modulates negative emotion processing mainly through reduced activity of regulatory brain regions in women, but not in men.
